# Editorial

**Published:** 1850-07

**Authors:** 


### VALEDICTORY ADDRESS.

BY S. P. HULLIHEN, M. D., D. D. S.

DELIVERED BEFORE THE GRADUATING CLASS OF THE BALTIMORE COLLEGE OF DENTAL SURGERY, 1849-50

The Dr. in this address gives some excellent advice to the graduating class—takes, as we think, a just view of the difficulties which hemmed in for a long time the progress of dental science, and closes with an eloquent appeal to maintain unstained the honors just attained. We give, however, an extract, which shows the difficulties out of which the profession has had to be extricated.

"Until within the last ten years—until the establishment of this College—the dental profession was looked upon as a *trade*, and its practitioners as mere mechanics; while gentlemen who devoted themselves to the treatment of the eye, the ear or skin, took rank at once with the physician or general surgeon. On what ground was this distinction predicated? By what authority was it sanctioned, and by whom promulgated? A disgraceful ignorance of medical science among dental practitioners was the ground-work. The medical faculty were the willing accusers, and the untiring persecutors.

They condemned, without stint, a calling they knew not how to practice, aud a practice they knew not how to improve. Such of the faculty as were learned in their profession, were found always competent and fully prepared, to be Oculists, Aurists, or Lithotomists, or to devote themselves to any other branch of the profession which their interest, inclination, or talents might determine, *except* that of dental surgery. This branch seemed to require something more than medical knowledge. It required great mechanical skill—"an education of the hand as well as of the head." A kind of education they had not received, and knew not where to acquire, and yet affected to despise. The necessities of the community cried aloud to them for help—a help which they could not bestow. This drove many sufferers to seek dental aid out of the medical profession, and to obtain that help which mechanical genius alone could supply. At this the profession seemed mortified and chagrined, and loudly mocked at those who dared to supply their delinquencies; and united as one man in deriding the uneducated dental mechanic.

They first created the necessity of an empiric, and then croaked forth their withering contempt on the creature their own ignorance had made."

The entire address is well written, and breathes the right spirit, and we are told was happily delivered.—J. T. Cin. Ed.

---

### DR. E. TOWNSEND'S ADDRESS.

We acknowledge the receipt in pamphlet form, of an excellent address, delivered by Dr. E. Townsend to the graduating class of the Baltimore College Dental Surgery, at their tenth annual commencement.

The address contains much good advice, which, if lived up to, must be of essential service to the gentlemen for whom it was designed, and is applicable to all. We make an extract from this admirable address, which we think is particularly appropriate to the student of dentistry or the young practitioner.

"Close application to study, and severe confinement at work, by depriving some of the functions of their due activity, and exhausting the energy of others, often induce a lassitude and inquietude to which narcotic stimulants afford a transient but delusive relief.

Intoxicating liquors are sometimes employed; tobacco still more frequently and incautiously, by professional men. Especially during the period of their preparatory studies, the weed is resorted to.

I intend to excuse myself for not preaching you a temperance sermon, and you must excuse *me*, gentlemen, for saying, avoid tobacco, as you respect both duty and decency. I do not stop to choose *terms*, for I can make no *terms* with the practice of using narcotics, and especially the most disagreeable of them. A few weeks since I observed a physician who only one year ago broke away from the habit of chewing, dexterously defend himself from the offensive breath of an entertaining friend, by twirling a chair on one of its legs between them as they stood together for a few last words before parting. As soon as the door closed upon the nuisance, he exclaimed, "Good Heavens! is it possible that I ever so annoyed any body while I used tobacco myself?" In an omnibus with all the windows open, I suffer from the effluvium which hangs about the clothes of smokers. What must patients in the chair suffer from the operator steaming the odious smell under their noses by the hour! And how greatly the indecorum is aggravated by the fact that the great majority of those who are thus oppressed, are *ladies!* to whom the dentist is quite dreadful enough for necessary inflictions, without being inexcusably disagreeable besides!!!"

---

We are truly glad to see that so many of our leading dentists at the east, are giving their countenance and support to our eastern Institution. Dental Colleges must soon take the same relative place in dental education, which is now occupied by our best medical schools. True, we hear of many dental practitioners who are yet opposed to all else but office instruction, and wish but little of that. We also hear of a few medical practitioners who take the same course, and *can recollect* when many did. They are alike in both cases, and posterity will do them justice.—J. T., Cin. Ed.

---

### PENNSYLVANIA SOCIETY OF DENTAL SURGEONS.

A SPECIAL meeting of this Society was held June 25th, 1850, at which was submitted a report on Evans' Amalgam, which we have given in the present number of the Register. We commend the spirit of the resolution submitted by the committee and accepted by the society.

The committee on Teeth made also a report. The sum and substance of which is, the award of a gold medal, worth twenty-five dollars, to Messrs. Jones, White, & Co., for the "*most improved artificial teeth.*"

These gentlemen appear to be doing quite a business in this way, and we are truly glad to see the spirit with which they enter the list. We hope some of these premium patterns will be sent to this city. We hope the competition between these gentlemen, Mr. Stockton and Mr. Alcock, will be such as to perfect this important branch of mechanical dentistry. We far prefer to see it take this course to that of a reduction of price. We think, however, that gum teeth, from the increased quantity now used, should be afforded for twenty cents, yet, rather than get them of an inferior quality, we would cheerfully give fifty cents, or even a dollar. Naturalness of form, perfectness of color, and strength, are the great desiderata; without these, any price is enough. We are happy, however to bear our testimony to the improvements which have been made by all these gentlemen. We hope soon to see some of the medal teeth, when we can speak more definitely of them.—J. T., Cin. Ed.

### DENTAL REGISTER.

The present numbei completes the third volume of this work. We owe an apology for the late date of this number, but our absence from the city, the prevalence of the epidemic, and the illness of a brother, must be our excuse. We would take this occasion to tender our thanks to those of the profession who have lent a helping hand, and by the prompt payment of their subscription, and otherways aided in this (as we think) much needed work. We would also remind others that they are behind in their dues, and that the trifle to them would be small indeed, yet when multiplied by fifty or sixty, it amounts to something, and that is needed to make the work what it should be. The Mississippi Valley alone should supply to a work of this kind three hundred subscribers. Indeed, every man who pretends to practice should take one or more periodicals which treat of his profession. The excuse that there is nothing new—it is all old to us, &c., &c.—is all downright foolishness, and no man of sense would make it. Such men are generally the veriest ignoramuses which degrade the profession.

The east, with the aid of the west, sustain their works devoted to dental science: should not the west, with the aid from the east, sustain one? Speaking of eastern aid, however, we would remind our eastern brethren that three or four subscribers are all we have from the great city and state of New York; Cincinnati alone does as well as this for all of the eastern publications. We know more subscribers in the State of Ohio to any of the eastern publications, than the entire "*Old Thirteen*" gives to the Dental Register. We are told that in New York there are some three hundred dentists; we would like to know if one-third take any work at all.

We are glad to say that, so far as we know, almost all in this city take some one of the publications. We know of no exceptions, save among some of the cheap concerns—half-price dentists. These have now, and perhaps always will have, a good excuse.—J. T. Cin. Ed.

### MISSISSIPPI VALLEY ASSOCIATION OF DENTAL SURGEONS.

This association is to convene on the second Tuesday of September next, at 10 o'clock, A. M., in the lecture room of the Ohio College of Dental Surgery, Cincinnati. We hope that all the members will be present, and make this one of the most profitable and pleasant meetings we have ever had. We are sure that three or four days could not be spent more pleasantly than the time consumed last year at the meeting of this Society.—J. T. Cin. Ed.

---

### OHIO COLLEGE OF DENTAL SURGERY.

The annual announcement of this Institution has been deferred, owing to the decease of J. T. Shotwell, M. D., Professor of Anatomy and Physiology. The Chair is now filled by T. Wood, M. D., a gentleman ardently devoted to this department of Medical Science, and brings with him an already acquired reputation as a surgeon. To give the student increased facilities for the aquisition of practical knowledge, an additional chair has been added—that of Mechanical Dentistry; and Dr. Leslie (who has for the past two years been demonstrator of this department, and who has discharged his duties to the general satisfaction of the class) been appointed Professor. Dr. King is every way qualified for the department over which he presides.—J. T., Cin. Ed.

---

### HARRIS'S PRINCIPLES AND PRACTICE OF DENTAL SURGERY.

The fourth edition of this valuable work is just from the press of Lindsay & Blackiston, Philadelphia. It is gotten up with their well known taste, and the fact that it has already passed to its fourth edition, speaks more for the value of the work, and its general appreciation, than all we could say—we have not had time to look through the work.

We draw attention to the Card of J. B. Dunlevy, Pittsburgh. We have had one sample of his gold foil, which we like very well. Several of our friends speak in the highest terms of it.

Mr. Dunlevy will attend to all orders promptly.—J. T., Cin. Ed.

---

### OBITUARY.

We regret to announce the decease of our colleague, Prof. Shotwell, M. D., who was Professor of Anatomy and Physiology in the Dental College, as well as in the Medical College of Ohio. Prof. Shotwell was an able and pleasant lecturer—kind and social—his warmth of feeling and manifest interest in the students' welfare, always made him popular with the class. His death will long be severely felt by his friends and the profession, of which he was an honorable member. Dr. Shotwell deceased Tuesday, 23d July, 1850.—J. T., Cin. Ed.

---

### ERRATA.

Page 162, 13 lines from bottom, for *this* read *the.*
" " 16 " " " *cooked* read *cooled.*
" 178, 12 " top " *action* read *active.*

---

The article on "snuff chewing," and a mode in which it may affect the pulmonary organs, by R. A. Kinloch, M. D., should have been accredited to the Charleston Medical Journal and Review.—J. T., Cin. Ed.

